# Traditional Chinese Medicine in the Treatment of Chronic Kidney Diseases: Theories, Applications, and Mechanisms

**DOI:** 10.3389/fphar.2022.917975

**Published:** 2022-07-18

**Authors:** Yunlai Wang, Ye Feng, Manman Li, Mo Yang, Gaoxiang Shi, Zihua Xuan, Dengke Yin, Fan Xu

**Affiliations:** ^1^ School of Pharmacy, Anhui University of Chinese Medicine, Hefei, China; ^2^ Anhui Province Key Laboratory of Chinese Medicinal Formula, Hefei, China; ^3^ Institute for Pharmacodynamics and Safety Evaluation of Chinese Medicine, Anhui Academy of Chinese Medicine, Hefei, China; ^4^ Scientific Research and Technology Center, Anhui University of Chinese Medicine, Hefei, China

**Keywords:** chronic kidney disease, renal fibrosis, traditional Chinese medicine, Chinese materia medica, reinforcing deficiency and purging excess, intervention mechanism

## Abstract

Chronic kidney disease (CKD) is a common and progressive disease that has become a major public health problem on a global scale. Renal fibrosis is a common feature in the pathogenesis of CKD, which is mainly related to the excessive accumulation and deposition of extracellular matrix caused by various inflammatory factors. No ideal treatment has yet been established. In recent years, based on the traditional Chinese medicine (TCM) theory of CKD and its molecular mechanism, clinical evidence or experimental studies have confirmed that a variety of Chinese materia medica (CMM) and their effective components can delay the progress of CKD. TCM believes that the pathogenesis of CKD is the deficiency in the root and excess in the branch, and the deficiency and excess are always accompanied by the disease. The strategies of TCM in treating CKD are mainly based on invigorating Qi, tonifying the kidneys, promoting blood circulation, removing stasis, eliminating heat and dampness, removing turbidity, and eliminating edema, and these effects are multitargeted and multifunctional. This review attempts to summarize the theories and treatment strategies of TCM in the treatment of CKD and presents the efficacy and mechanisms of several CMMs supported by clinical evidence or experimental studies. In addition, the relationship between the macroscopic of TCM and the microscopic of modern medicine and the problems faced in further research were also discussed.

## Introduction

In recent years, research on the mechanism and intervention strategies of chronic kidney disease (CKD) has become a hot spot in the nephrology field. CKD is defined by the Kidney Disease Outcomes Quality Initiative in terms of either kidney damage or decreased glomerular filtration rate (GFR, <60 ml/min per 1.73 m^2^) with or without evidence of kidney damage, for three or more months, regardless of the cause (National Kidney Foundation, 2002; Steven and Levin, 2013). It is characterized by increased inflammatory cell infiltration, tubular atrophy, tubulointerstitial fibrosis, and glomerulosclerosis, finally leading to some forms of end-stage renal disease (ESRD) or renal failure (Boor et al., 2010). However, in ESRD, the survival of patients depends on the renal replacement therapy or dialysis because of lack of kidney donors. The global burden of CKD study in 2017 showed that the global prevalence of CKD has exceeded 9%, accounting for 18.97% of CKD patients worldwide living in China (GBD Chronic Kidney Disease Collaboration, 2020). In 2017, CKD resulted in 1.2 million deaths, and the number has been projected to rise to 2.2 million (best-case scenario) and up to 4.0 million (worst-case scenario) by 2040 (Foreman et al., 2018). Therefore, CKD has become a major public health problem on a global scale. Delaying or preventing the progress of CKD has become an important challenge facing the clinical medicine community and the health departments of various countries.

Current therapy for CKD includes angiotensin-converting enzyme inhibitors and angiotensin-receptor blockers, which act by decreasing proteinuria, lowering blood pressure, and thus retarding CKD progression (Levey and Coresh, 2012; Ruggenenti et al., 2012). However, the treatments are not sufficient for all patients and long-term medication may lead to a number of adverse effects such as hyperkalemia and acute kidney injury (Kidney Disease Outcomes Quality Initiative (K/DOQI), 2004). As an important branch of complementary and alternative medicine, traditional Chinese medicine (TCM) has been proved to protect people’s health for thousands of years. Preclinical studies or clinical trials have shown that Chinese materia medica (CMM), a form of TCM treatment, is promising in treating CKD, especially in the aspects of reducing proteinuria and adverse effects of western drugs, and reduces ESRD risk by 60% (Wojcikowski et al., 2006; Lin et al., 2015). As a large number of people use herbs for medicinal purposes, the safety of CMM has been questioned. The most well-known adverse effect is nephropathy induced by aristolochic acid, which resulted in ESRD and urothelial malignancy. The mechanism of nephrotoxicity induced by aristolochic acid has been clarified as mainly related to the induction of tubular cell apoptosis, the formation of aristolochic acid-DNA adducts, and the inhibition of mitochondrial ATP synthesis (Nortier and Vanherweghem, 2007; Han et al., 2019). Some studies suggest that nephrotoxic effects may be caused by incorrect use of CMMs or toxic CMMs or potentially toxic CMMs (Jha et al., 2013; Jha and Rathi, 2018). It is important to point out that the use of CMMs by physicians practicing TCM is based on the theory of TCM and they prescribe formulas on the basis of syndrome differentiation and treatment approach (Li L. et al., 2019). According to the ancient compatibility rule of “Jun-Chen-Zuo-Shi” (“monarch-minister-assistant-courier”), making a prescription with two or more CMMs can increase the medicinal effects and restrain the CMM’s toxic properties (Wei and Zheng, 2008). Therefore, the syndrome differentiation and treatment approach and compatibility theory ensure the efficacy and safety of CMMs.

To date, the pathophysiological mechanism of CKD has been reviewed in some studies (Chen et al., 2018; Lv et al., 2018; Rinschen and Saez-Rodriguez, 2021). Some evidence has shown that single CMM and CMM formulas possess a range of important pharmacological properties in improving CKD. In this review, we attempt to discuss the current knowledge of TCM for the treatment of CKD and its possible interventional mechanisms.

## Understanding of CKD in TCM

The advantages of TCM in the treatment of CKD are mainly reflected in the overall concept and syndrome differentiation. The concept of organs in TCM is different from that in modern medicine. As recorded in the “Yellow Emperor’s Canon of Internal Medicine,” the kidney is the place of true Yin and true Yang and the base of hiding and the place for storing the essence. In the TCM theory, the essence transforms Qi (the vital energy) and produces Blood (the body circulation) (Tu X. et al., 2013). Therefore, abnormalities of the kidney are believed to cause the disorder of the body. TCM classifies CKD into the categories of “edema,” “retention of urine,” and “kidney fatigue.” As CKD is characterized by severe proteinuria, the lesions are mainly in the spleen and kidneys. Proteinuria is the pathological product of human essence substance flowing down and leakage of the essence. A basic substance that constitutes the human body and sustains life activities is similar to “vital essence” in TCM, which emphasizes that these essences should be stored in the human body and should not be released. Damage to the kidney leads to a loss of vital substances, resulting in the deficiency of Qi and Yang. Furthermore, the kidney is called the viscera of water, and it is responsible for the body fluid. If the water is stagnant, dampness and heat will occur (Ding et al., 2014). Moreover, with the damage to the kidney essence, Qi cannot consolidate the Blood, resulting in unfavorable blood circulation and blood stasis (anticoagulation) formation (Li X. et al., 2019a).

Besides, the vital substances stored in the kidneys depend on the transportation and distribution of the spleen. The disorder of spleen transport is usually caused by improper diet, deficiency of endowment, and excessive fatigue, which lead to spleen Qi deficiency. Furthermore, with the development of spleen Qi deficiency, the development of disease, the decline of fire in the vital gate, and the loss of warm spleen, it will further lead to the deficiency of spleen Yang. The spleen cannot warm the grain and liquid, and then the transport of liquid and water is abnormal, which leads to water dampness. As mentioned in “Plain Questions,” with Yin and Yang in relative balance, the spirit can be cured. Thus, TCM believes that “spleen-kidney deficiency” is an internal condition; blood stasis, internal dampness, and heat are inextricably linked to the patient’s viscera (Shi and Shen, 1982; Nie, 2008; Wu and Ma, 2011; Su et al., 2013). The treatment principle for CKD is reinforcing deficiency and purging excess, and simultaneous treatment of the branch and the root to achieve “Yin and Yang in relative balance” (stabilization status).

The pathogen of CKD is mainly manifested as blood stasis, dampness heat (hygropyrexia), and turbid toxin (retained hazardous substances). Blood stasis syndrome is one of the most common CM syndromes among patients with primary glomerular disease (Li et al., 2009). “Blood stasis” in TCM covers glomerulosclerosis, an increase of the extraglomerular matrix, thickening of the basement membrane, adhesion of balloon, microthrombosis in the glomerulus, collapse or stenosis of the capillary lumen, compression and occlusion of vascular loops, and tubulointerstitial fibrosis and atrophy (Wu and Ma, 2011; Guo et al., 2019). TCM has the viewpoint that by removing excessive patterns of stagnated blood, the Blood and Qi can be invigorated, and then they promote blood circulation. This condition is suitable for promoting blood circulation and removing blood stasis, invigorating Qi to reduce swelling.

When the spleen and kidneys are deficient, water dampness is endogenous. Pathogenic dampness resides in the body, leading to heat from Yang, or heat from dietary intake, and eventually pathogenic dampness changes to heat from dampness. As the famous medical scientist Lingtai Xu inferred, there must be heat when there is dampness. In microscopic differentiation of syndromes, dampness heat in renal pathology often shows swelling of endothelial cells, formation of microthrombus, narrowing of the capillary lumen, and release of inflammatory mediators (Shen, 2008; Yu et al., 2016). The theory of lipid toxicity and abnormal hemodynamics can also refer to the syndrome differentiation of dampness heat. If the dampness heat is not treated, the mediating center function of the spleen and stomach will be further affected, the metabolites produced in the body will accumulate in the body, and the biological activity of many metabolites can further lead to the clinical uremic syndrome. These solutes are called uremic toxins, which are equivalent to the category of turbid toxin in TCM (Guo et al., 2019). Turbid toxin acts on the human body and leads to turbidities of cells, tissues, and organs, including hypertrophy, hyperplasia, atrophy, metaplasia, and canceration in modern pathology, as well as changes in inflammation, degeneration, apoptosis, and necrosis (Wang and Zhang, 2003; Wang Z. et al., 2010). The relationship between dampness heat and turbid toxin can be summarized as follows: dampness is the source of turbidity, turbidity is the further step of dampness, heat is the gradual toxin, and the toxin is the extreme of heat. It is important to promote the excretion or removal of dampness heat and turbid toxin in CKD, using methods such as the method of clearing heat, dispelling dampness, and removing turbidity.

## TCM for Treating CKD

**FIGURE 1:**
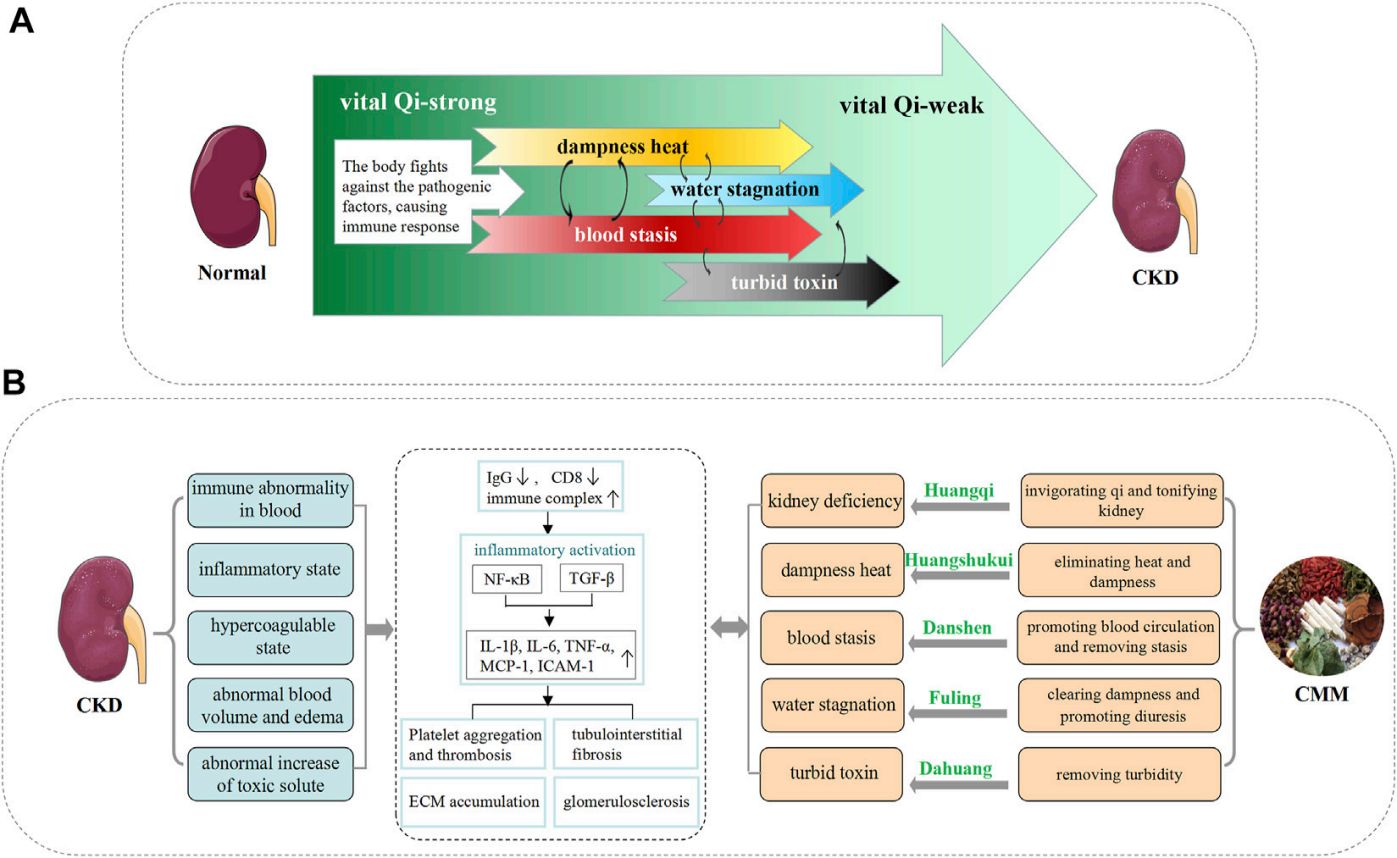
The corresponding relationship between the pathogenesis of the TCM theory and the modern medicine theory. **(A)** Characteristics of TCM pathogenesis in the process of chronic kidney disease. In the early stage of CKD, although the spleen and kidneys are insufficient, the vital Qi can still resist exogenous pathogens, and the pathogenesis is characterized by pathogenic excess (such as dampness heat and blood stasis). However, as the disease progresses to the middle stage, the vital Qi cannot overcome the pathogenic factors, various pathological products are formed, and its pathogenesis characteristics change into a mixture of deficiency and excess. When the disease progresses to the terminal stage, the vital Qi is exhausted, and the pathogenesis is characterized by a deficiency of vital Qi (leading to the accumulation of liquid and turbid toxin). In different stages of nephropathy, the priority of the deficiency and the excess are different. Deficiency and excess are cause and effect of each other, which leads to the continuous progress of CKD. **(B)** Cognition and treatment strategy of CKD in the TCM theory. On the left are the modern medical characteristics and the main pathological process of CKD. On the right is the treatment strategy of TCM using CMM, and the representative CMMs corresponding to each pathogenesis are listed.

According to the long-term clinical practice, different CMMs have different pharmacological effects and are endowed with different TCM efficacies. Previous studies have shown that in patients with CKD stage 3 with different TCM syndrome patterns, four different therapies including invigorating Qi and Blood, promoting blood flow, expelling wind-evil (a kind of exogenous pathogenic factors), and clearing heat and dissipating dampness (regulating the immune system and promoting urination) significantly improved the glomerular filtration rate and decreased proteinuria (Wang YJ. et al., 2012). This reflects the basic principle that TCM adopts different treatment strategies for different syndromes (Figure 1). Here, we summarize the strategies and mechanisms of TCM against CKD from different forms of medication, such as single CMM or CMM extracts, CMM pairs, and CMM formula.

### Single CMM or CMM Extracts

The difference between TCM and modern medicine is that it has the significant property of being multicomponent. Single CMM is a unit that contains the least amount of components in TCM, and the pharmacodynamic material basis has been studied most deeply. With the continuous progress of research, the mechanism of single CMM or CMM extracts for CKD is gradually been clarified.

#### Astragali Radix for Invigorating Qi

Astragali Radix (AR, Huangqi) is derived from the dry roots of *Astragalus membranaceus* (Fisch.) Bge. var. *mongholicus* (Bge.) Hsiao or *Astragalus membranaceus* (Fisch.) Bge. In the clinical practice of TCM, AR is mainly used to invigorate Qi, manifested in two aspects, supplementing the Defense-Qi (which has the physiological functions of defending exogenous pathogenic factors, warming and nourishing the whole body) and replenishing the Middle-Qi (which is the function motivity of spleen and stomach) (Chen Z. et al., 2020). Therefore, as recorded in “Compendium of Materia Medica,” AR is known as the key CMM of tonics. The chemical studies of its ingredients are relatively clear, mainly including triterpenoid saponins (mostly cycloartane-type), flavonoids, and polysaccharides (Su et al., 2021).

The effects of AR on the reduction of proteinuria and serum creatinine have been studied in patients. After treatment with AR injection in 30 patients with chronic glomerulonephritis, the content of urine protein decreased obviously from 2328 ± 3157 to 1017 ± 765 mg/24 h (Shi et al., 2002). The effect of AR on reducing total cholesterol, triglyceride, and low-density lipoprotein (LDL) and increasing plasma total protein and albumin has also been confirmed in patients with CKD (Zhang et al., 2005). In addition to small clinical trials, a systematic review of 66 studies involving 4,785 diabetic kidney disease participants also demonstrated the benefits of AR injection or preparation in reducing albuminuria, proteinuria, and serum creatinine levels (Zhang L. et al., 2019). Furthermore, AR-containing preparations are effective in improving eGFR in patients with mild to moderate CKD (Yoshino et al., 2022).

In recent years, the immunomodulatory effect of AR has been gradually developed (Wang YP. et al., 2002; Chen Z. et al., 2020). A previous study has shown that AR significantly downregulated CD_4_ and upregulated CD_8_ in patients with chronic glomerulonephritis, suggesting that it has a regulatory effect on cellular immunology (Shi et al., 2002). Several pieces of evidence have accumulated showing the potential of AR in reducing water retention (Ma et al., 1996; Wang H. Y. et al., 2002). The therapeutic effect of AR on nephrotic syndrome (NS) may be demonstrated by reducing the expressions of arginine vasopressin (AVP) mRNA, AVP V2 receptor mRNA, and AVP-dependent aquaporin-2 (AQP2) mRNA, thereby eliminating edema. In addition, AR has the effect of ameliorating the blunted renal response to the atrial natriuretic peptide (Ma et al., 1999). It suggested that AR is different from diuretics in the treatment of edema. AR protects against oxidative stress-induced damage in proximal tubular epithelial cells *via* antiapoptotic and antiinflammatory mechanisms (Shahzad et al., 2016). It was demonstrated by the downregulation of pro-apoptotic Bax and upregulation of Bcl-XL, a decrease in nuclear factor kappa B (NF-κB, p65, p50), a decrease in tumor necrosis factor (TNF)-α, and an increase in transforming growth factor (TGF) β1.

As the main active ingredient of AR, astragaloside significantly improved the state of oxidative stress in podocytes cultured with adriamycin (ADR) and suppressed the cytoskeletal rearrangement, improving the migration ability of podocytes *via* upregulating the expression of matrix metalloproteinase 2 (MMP-2) and MMP-9 (Sai et al., 2018). The most studied astragaloside IV can inhibit high glucose-induced cell apoptosis through increasing hepatocyte growth factor production *via* its specific receptor c-met and inhibiting the phospho-p38 mitogen-activated protein kinase (MAPK) signal pathway (Mou et al., 2008; Wang Q. et al., 2014). Calycosin is another component of AR that can inhibit the phosphorylation of IKBα and NF-κB p65 in db/db mice and cultured mouse tubular epithelial cells (Zhang YY. et al., 2019).

#### Rhei Radix et Rhizoma and Abelmoschi Corolla for Eliminating Heat and Dampness

Rhei Radix et Rhizoma (RRR, Dahuang), the dried roots and rhizomes of *Rheum palmatum* L., *Rheum tanguticum* Maxim. Ex Balf., or *Rheum officinale* Baill, is widely used as a laxative for many years. Pharmacological studies have confirmed its multiple activities, such as antibacterial, purgative, antifibrosis, regulating gastrointestinal, antiinflammatory, and antitumor activities (Xiang et al., 2020). In a systemic review of 18 randomized or quasirandomized trials including 1,322 patients with CKD, RRR showed significant positive effects on relieving symptoms, lowering serum creatinine, and adjusting disturbance of lipid metabolism (Li et al., 2004). In addition, in a prospective clinical trial of 151 patients with chronic renal failure (CRF), the frequency of end-stage renal failure in the RRR group was 52% lower than that in the ACE inhibitor group. Besides, a slope of progression as the progressive rate of decline in renal function was found to be more horizontal in the RRR-treated group (Li, 1996).

As shown by Zhang et al., treatment with the extracts of RRR reversed the abnormalities of the urinary metabolites in adenine-induced CKD animals and reduced the expression of histopathological inflammatory markers such as collagen (Col) I, Col III, and pro-fibrosis marker TGF-β1 (Zhang et al., 2015). Research on network pharmacology suggested that RRR could target multiple targets involved in the accumulation of extracellular matrix (ECM), the release of inflammatory factors, the balance of coagulation, and fibrinolysis, which showed a synergistic therapeutic effect (Xiang et al., 2015). Regarding the mechanism of RRR against renal fibrosis, some studies have shown that its nephroprotective effect was to reduce the expressions of TGF-β1, TGF-β receptor I (TGF-β RI), TGF-β RII, Smad2, p-Smad2, Smad3, p-Smad3, and Smad4, meanwhile increasing Smad7 (Zhang ZH. et al., 2018). In addition to improving renal fibrosis, RRR could also improve the intestinal barrier function in 5/6 nephrectomy (5/6Nx) rats by regulating systemic inflammation, intestinal barrier markers, and toll-like receptor 4-myeloid differential protein-88-NF-κB inflammatory response (Ji et al., 2020).

Rhein is the main active component of RRR. Recent studies have shown that in adenine mice or unilateral ureteral obstruction (UUO) mice, rhein effectively recovered Klotho promoter hypermethylation *via* reversing aberrant DNA methyltransferases expression, thus upregulating the expression of Klotho protein (Zhang et al., 2016; Zhang et al., 2017). Besides, the renal protective mechanism of rhein was also related to the activation of the sirtuin 3-forkhead box O3α signaling pathway to exert antioxidant capacity and the regulation of AMP-activated protein kinase (AMPK)/mammalian target of rapamycin (mTOR) signaling pathways to inhibit autophagy (Tu et al., 2017; Wu et al., 2020).

Abelmoschi Corolla (AC, Huangshukui), the flower of *Abelmoschus manihot* (L.) Medic., a single medicament of TCM for eliminating heat and dampness, has been widely used for the treatment of CKD in China. As a modern preparation extracted from AC, the Huangkui capsule has been approved by the China National Medical Products Administration (Z19990040). As reviewed by Sun et al., in approximately 2,000 CKD patients, AC showed to decrease proteinuria with stable kidney function during follow-up (Sun X. et al., 2022). Besides, after a comprehensive evaluation, the clinical value of the Huangkui capsule in treating CKD is class B and that for diabetic nephropathy (DN) and chronic nephritis is class A (Wang et al., 2022).

For the mechanistic studies, the Huangkui capsule has been shown to improve kidney inflammation and glomerular injury in ADR-induced nephropathy through inhibition of the p38 MAPK signaling pathway and AC inhibits ROS-ERK1/2-mediated NLRP3 inflammasome activation (Tu Y. et al., 2013; Li W. et al., 2019b). Recent research demonstrated that Huangkui capsule protection against renal fibrosis is dependent on the transient receptor potential channel 6 (TRPC6) pathway (Gu et al., 2020). In detail, the Huangkui capsule inhibited the expressions of a plethora of pro-inflammatory mediators in UUO mice by suppressing both canonical (Smad2/3) and noncanonical (MAPK) signaling pathways and the TRPC6/calcineurin A/nuclear factor of the activated T-cells signaling axis, which were possible through direct inhibition of TRPC6 activity in a heterologous expression system and indirect suppression of TRPC6 expression in both WT and TRPC6 knockout mice. Flavonoids, such as quercetin, isoquercitrin, hyperoside, gossypetin-8-O-β-D-glucuronide, and quercetin-3-O-glucoside, can inhibit the epithelial to mesenchymal transition in HK-2 cells (Cai et al., 2017). An et al. indicated that hyperoside pretreatment could significantly decrease albuminuria and prevent glomerular basement membrane (GBM) damage and oxidative stress in diabetes mellitus mice by decreasing podocyte heparanase expression (An et al., 2017). Liu et al. have revealed that quercetin ameliorated pyroptosis and injury in podocytes under HG conditions *via* adjusting METTL3-dependent m^6^A modification and regulating NLRP3-inflammasome activation and PTEN/PI3K/Akt signaling (Liu et al., 2021).

#### Salviae Miltiorrhizae Radix et Rhizoma for Promoting Blood Circulation and Removing Stasis

Salviae Miltiorrhizae Radix et Rhizoma (SM, Danshen) is a CMM derived from the root of *Salvia miltiorrhiza* Bunge. It exhibits various pharmacological activities, such as antioxidant activities, inhibiting the expression of adhesion molecules, antiplatelet aggregation, inhibiting mast cell degranulation, and inhibiting apoptosis and is commonly used for promoting blood circulation and removing stasis (Han et al., 2008). Previous studies have demonstrated that the injection of SM preparation could significantly reduce blood urea nitrogen and creatinine and improve renal function, probably by its antioxidant effects (You et al., 2012; Lu et al., 2015).

In the study of metabonomics in rats with CRF, 54 metabolites could be regulated by SM ethanol extract and water extract (Cai et al., 2018). Moreover, the extract of SM mentioned above significantly regulates the expressions of alpha-smooth muscle actin (α-SMA), FN, E-cadherin, p-ERK, NADPH1 oxidase 1 (NOX1), NOX2, NOX4, TGF-β, TGF-βRI, TGF-βRII, Smad2, Smad3, and Smad7 in HK-2 cells, that is, modulating the NADPH oxidase/ROS/ERK and TGF-β/Smad signaling pathways. A recent study also showed that SM can significantly regulate intestinal bacteria in CRF rats.

Several components isolated from SM have been used to treat kidney disease. The pharmacodynamic effects of salvianolic acid A on 5/6Nx rats showed that it could reduce proteinuria, improve renal function, and reduce renal tubulointerstitial fibrosis. The mechanism is closely related to its antiinflammatory activities through inhibition of the activation of the NF-κB and p38 MAPK signaling pathways (Zhang HF. et al., 2018). In addition, salvianolic acid A treatment improved glucocorticoid resistance of podocytes, partly by modulating the soluble urokinase plasminogen activator receptor (suPAR)/uPAR-αvβ3 signaling pathway (Li X. et al., 2021).

In general, the effect of SM on promoting blood circulation and removing stasis may be related to the increase in renal blood flow, the decrease of the expression of hypoxia-inducible factor (HIF)-1α and vascular endothelial growth factor (VEGF), and the recovery of expression of neuronal nitric oxide synthase (nNOS) by magnesium lithospermate B (Lin et al., 2019). On the other hand, the effects of magnesium lithospermate B on attenuating renal function, renal fibrosis, and inflammation were investigated in 5/6Nx rats, showing a reduction in the expressions of FN, Col III, Col IV, TNF-α, and monocyte chemoattractant protein-1 (MCP-1), which are associated with inhibition of TF activation (Wang et al., 2019). In addition, other studies have shown that inhibition of the mitochondrial apoptosis pathway, i.e., inhibition of mitochondrial Bax accumulation and release of cytochrome c, may also be the renal protective mechanism of magnesium lithospermate B.

#### Poria for Clearing Dampness and Promoting Diuresis

Poria (Fuling) is the dry sclerotium derived from *Poria cocos* (Schw.) Wolf (Polyporaceae). The inner parts of Poria are commonly known as “Fuling” in Chinese. Triterpenoids are the main components of Poria, and it exhibits a variety of biological activities such as antiinflammatory, immunomodulatory, antitumor, antioxidant, and antibacterial activities (Ríos, 2011; Miao et al., 2016; Li X. et al., 2019). In TCM, Poria has the effects of strengthening the spleen, promoting diuresis, and eliminating edema and is mainly used to treat the disorder of fluid metabolism. In the ADR-induced NS rat model, Poria extract could significantly improve the levels of urine protein, creatinine, serum total cholesterol, and IL-4 cytokine and attenuate kidney injury (Zan et al., 2017). Likewise, Poria treatment significantly improves the disturbance of water metabolism in puromycin aminonucleoside (PAN)-induced NS rats by inhibiting the expression of the epithelial sodium channel and AQP2 (Lee et al., 2014). An *in vitro* study also revealed that Poria treatment inhibited the activation of Sgk1 and decreased the expression of TonEBP mRNA (Lee et al., 2012). Its effect on AQP2 might be related to the inhibition of PKA and the decrease of the cAMP content.

Because of the obvious diuretic effect of ethanol extract from the surface layer of Poria, Zhao et al. focused on the protective effect of triterpenoids from the surface layer of Poria on CKD (Zhao et al., 2012). Several studies have demonstrated that triterpenoids isolated from the surface layer of Poria have beneficial renal protective effects on renal fibrosis. Poricoic acid A is the main triterpenoid in the surface layer of Poria. It exhibited the suppression of fibrosis by stimulating AMPK and inhibiting Smad3, preventing abnormal ECM accumulation and remodeling, and promoting the deactivation of fibroblasts (Chen D. Q. et al., 2020). Poricoic acid A combined with melatonin has a protective effect on acute kidney injury (AKI)-to-CKD transition. On the 14th day of renal ischemia-reperfusion injury in rats, poricoic acid A and melatonin treatment significantly inhibited the expressions of α-SMA, Col I, and FN. Inhibition of the TGF-β/Smad, Wnt/β-catenin, and NF-κB/Nrf2 pathways may be the mechanisms of poricoic acid A against renal fibrosis (Chen D. Q. et al., 2019a; Chen D. Q. et al., 2019b). Interestingly, poricoic acid A and melatonin could upregulate growth arrest-specific 6 (Gas6)/Axl signaling to reduce oxidative stress and inflammation in the AKI disease status and further downregulate Gas6/Axl signaling to attenuate renal fibrosis in the CKD disease status (Chen D. Q. et al., 2019b). This may reveal the bidirectional regulation of TCM to some extent. As reported previously, some new poricoic acids such as poricoic acid ZC, poricoic acid ZD, poricoic acid ZE, poricoic acid ZG, poricoic acid ZH, poricoic acid ZI, poricoic acid ZM, and poricoic acid ZP significantly attenuated renal fibrosis though the same mechanisms as poricoic acid A *in vivo* and *in vitro* (Wang et al., 2018a; Wang et al., 2018b; Chen L. et al., 2019; Wang M. et al., 2020a).

### CMM Pairs

CMM pairs (combination of two CMMs) are the most basic composition units, the simplest form of multi-CMM formula, and a centralized representative of CMM compatibility (Wang S. et al., 2012). It is not the superposition of any two CMMs but contains the wisdom and clinical experience of TCM doctors.

#### Astragali Radix-Angelicae Sinensis Radix Pair for Invigorating Qi and Promoting Blood

AR and Angelicae Sinensis Radix (AS, Danggui, the root of *Angelica sinensis* (Oliv.) Diels) are a common CMM pair used to treat kidney disease for years. The combination of the two CMMs is also known as Danggui Buxue Tang (DBT) with a weight ratio of 5:1. When the two CMMs are decocted together, it can help a better dissolution of effective components and the generation of new components. For example, with the increase in the ratio of AR, the dissolution of ferulic acid and ligustilide increased (Gao et al., 2007). Moreover, the results indicated that immunomodulatory, oesteotropic, and estrogenic effects were best exerted at the AR to AS ratio of 5:1.

According to what has been mentioned before, blood stasis always accompanies CKD. Studies have shown that the AR–AS pair also could promote recovery of blood flow, improve the microstructure dysfunction, enhance NO production *via* activating eNOS, and induce erythropoietin (EPO) mRNA expression and secretion of EPO protein (Meng et al., 2007; Gao et al., 2008). The mechanism acting on EPO might be related to increasing the expression of HIF-1α mRNA and promoting the protein translation of HIF-1α *via* a Raf/MEK/ERK-dependent signaling pathway (Zheng et al., 2010). However, some studies reported that the CMM pair could not reduce urinary protein (Lu et al., 1997; Li et al., 2000). It was suggested that the effect of the pair on preventing and treating glomerulosclerosis might not directly affect hemodynamics or filtration membrane permeability but is related to regulating lipid metabolism by upregulating the expression of hepatic LDL receptor gene and increasing the activities of serum lipoprotein and lecithin-cholesterol acyltransferase.

The immunomodulatory effect of DBT is reflected in the immune activation in human T-lymphocytes and macrophages. Specifically, DBT could significantly induce the proliferation of cultured T-lymphocytes and the secretion of IL-2 and p-ERK and increase the phagocytosis of cultured macrophages (Gao et al., 2006). This is consistent with the view of the TCM theory that TCM with beneficial Qi participates in improving the defense ability of the immune system.

The antirenal fibrosis effect of the AR–AS pair has been extensively studied, and its mechanism mainly includes downregulating the expressions of TGF-β1 and connective tissue growth factor (CTGF), preventing the activation of c-Jun N-terminal kinase (JNK)-MAPK, and inhibiting the expression of NLRP3 inflammasomes (Huang et al., 2003; Wang et al., 2003; Wang et al., 2016). Integrated lipidomics, transcriptomics, and network pharmacology techniques were also used to reveal the mechanisms of DBT (Sun L. et al., 2022). Just as the AR–AS pair, astragaloside IV and ferulic acid have been proved to improve renal tubulointerstitial fibrosis and protect the function of vascular endothelium, which may be the pharmacological material basis of the pairs (Meng et al., 2011; Yin et al., 2014).

#### AR and Notoginseng Radix et Rhizoma Pair for Invigorating Qi, Promoting Blood Circulation, and Removing Stasis

Sanqi oral solution (SQ), composed of AR and Notoginseng Radix et Rhizoma (Sanqi, the roots and rhizomes of *Panax notoginseng* (Burk.) F.H.Chen), is a patented hospital preparation, which was designed and developed by the famous nephrologist Nizhi Yang (Tian et al., 2019). Notoginseng Radix et Rhizoma is an important CMM related to Blood, with the features of promoting blood circulation, removing blood stasis, inducing blood clotting, and alleviating pain (Lei and Chiou, 1986; Cicero et al., 2003). Based on the efficacy of AR in invigorating healthy Qi and Notoginseng Radix et Rhizoma in eliminating pathogenic factors, the combination has been used for clinically treating CKD for over 20 years with good curative effect. In a rat model of membranous nephropathy, SQ successfully reduced proteinuria, increased serum albumin, and restored podocyte injury, which was associated with the inhibition of the NF-κB signaling pathway (Tian et al., 2019). Moreover, SQ could also exert renoprotective effects on renal I/R injury by inhibiting apoptosis and enhancing autophagy, combined with regulating ERK/mTOR pathways (Tian et al., 2020).

Since both have a distinct ability to regulate T-lymphocyte subsets (Yang Z. et al., 2007; Wan et al., 2013), SQ indicated regulation of lymphocyte subsets and reduction of macrophages infiltration in renal tissues (Wei et al., 2013). *In vitro* experiments showed that inhibition of mesangial cell proliferation by SQ may be related to inhibition of IL-β1 secretion and gene expression and promotion of IL-10 secretion and gene expression (Yang N. Z. et al., 2007). Thus, it was considered that mesangial cells were important target cells of SQ in preventing and treating chronic nephritis and delaying CKD progression.

### CMM Formula

CMM formula is the main form of clinical treatment of TCM, which is composed of two or more CMMs based on the theory of TCM. Under the appropriate dosage ratio, many CMMs in the formula can make full use of their advantages, avoid their disadvantages and play a synergistic role to realize the overall adjustment of TCM. CMM pairs are the smallest component units of a formula, and some representative CMM pairs have been described above. This section mainly discusses the characteristics of CMM formulas composed of multiple CMMs in the study of CKD and the details are summarized in Table 1.

**TABLE 1:** Characteristics of TCM formulas on CKD.

Formulas	TCM efficacy	Composition	Model/cell type	Mechanism	Ref.
Bu-Shen-Huo-Xue formula	Tonifying kidneys, nourishing Qi, and invigorating blood circulation	Astragali Radix, Curcumae Rhizoma, Rhei radix et Rhizoma, Trigonellae Semen, Vaccariae Semen, Smilacis Glabrae Rhizoma, and Coptidis rhizoma	5/6 Nx rats	↓TNF-α, CTGF, TGF-β1, NF-κB, and OPN; ↑PPAR-γ	Lu et al. (2014)
Fuzheng Huayu recipe	Strengthening the body resistance and removing stasis	Cordyceps, Salviae Miltiorrhizae Radix et Rhizoma, Persicae Semen, Schisandrae Chinensis Fructus, Pini Pollen, and Gynostemma Pentaphyllammak	HgCl_2_-RIF rats	↓TGF-β1, p-Smad2, p-Smad3, TβR-I, miR-21, and AKT; ↑E-cadherin and PTEN	Qing-Lan Wang et al. (2010); Qing-Lan Wang et al. (2020)
Fangji Huangqi Tang	Nourishing Qi, strengthening the spleen, and promoting diuresis	Astragali Radix, Stephaniae Tetrandrae Radix, Atractylodis Macrocephalae Rhizoma, and Glycyrrhizae Radix et Rhizoma	ADR-NS rats	↑Podocin	Yu et al. (2009)
Huang Qi Huai granules	Improving Qi and nourishing Yin	Poria Robiniophila, Lycii Fructus, and Polygonati Rhizoma	ADR-NS rats	↑Nephrin; ↓TNF-α, NF-κB p65, IκBα, Bax, and caspase-3	Liu et al. (2017)
Shenyan Xiaobai granule	Invigorating the spleen and kidneys, clearing heat, and removing dampness	Astragali Radix, Smilacis Glabrae Rhizoma, Codonopsis Radix, Dioscoreae Rhizoma, Coicis Semen, Ligustri Lucidi Fructus, Cuscutae Semen, Rehmanniae Radix Praeparata, Lycii Fructus, Imperatae Rhizoma, Leonuri Herba, and Euryales Semen	ADR-NS rats	↑Nephrin and podocin	Cao et al. (2019)
Yishen Huoxue prescription	Improving Qi, activating Blood, and removing stasis and turbidity	Astragali Radix, Angelicae Sinensis Radix, Chuanxiong Rhizoma, Salvia Miltiorrhizae Radix et Rhizoma, Rhei Radix et Rhizoma, and Notoginseng Radix et Rhizoma	UUO rats	↑miR-126, VEGFA, VEGFR-2, and Notch1	Zhong et al. (2018)
You-gui pill	Warming and recuperating kidney-Yang	Rehmanniae Radix Praeparata, Dioscoreae Rhizoma, Corni Fructus, Lycii Fructus, Cuscutae Semen, Cervi Cornus Colla, Eucommiae Cortex, Cinnamomi Cortex, Angelicae Sinensis Radix, and Aconiti Lateralis Radix Praeparata	UUO rats and TGFβ1-stimulated NRK-49F cells	↓TGF-β1, α-SMA, FN, Col, and p-Smad2/3	Li Wang et al. (2015)
Yiqi Huoxue formula	Nourishing Qi, invigorating blood circulation, strengthening the spleen, and promoting diuresis	Astragali Radix, Angelicae Sinensis Radix, Chuanxiong Rhizoma, Salvia Miltiorrhizae Radix et Rhizoma, Paeoniae Radix Rubra, Plantaginis Herba, Achyranthis Bidentatae Radix, and Taraxaci Herba	UUO rats	↓TGF-β, smad2, and CTGF; ↑smad7	Liu et al. (2010)
Shen Shuai II recipe	Strengthening the spleen, tonifying kidney-Yang, activating blood circulation, and removing stasis	Salvia Miltiorrhizae Radix et Rhizoma, Epimedii Folium, Codonopsis Radix, Angelicae Sinensis Radix, Rhei Radix et Rhizoma, Perillae Folium, Persicae Semen, Chuanxiong Rhizoma, and Coptidis Rhizoma	5/6 Nx and infarcted rats and hypoxic NRK-52E cells	↓HIF-1α, c-Myc, IL-1β, Bax, Puma, p53, and cytochrome c	Meng Wang et al. (2020); Yang et al. (2021)
Shenkang	Improving Qi and removing stasis and turbidity	Rhei Radix et Rhizoma, Salvia Miltiorrhizae Radix et Rhizoma, Carthami Flos, and Astragali Radix	UUO mice, adenine-CRF rats, TGF-β1-stimulated NRK-49F cells, and TAC-NS rats	↓Prdx5, JAK2/STAT3, SOCS1, SOCS3, NF-κB p65, IκBα, COX-2, MCP-1, iNOS, and Keap1; ↑Nrf2, HO-1, catalase, and GCLC	Luo et al. (2021); Qin et al. (2021)
Nephrokeli	Nourishing kidneys	Ligustri Lucidi Fructus, Testudinis Carapax et Plastrum, Dioscoreae Rhizoma, Typhae Pollen, Ecliptae Herba, Perilla, Atractylodis Rhizoma, Coicis Semen, Rehmanniae Radix, and Hedyotis Diffusa	IgAN rats and S1P-stimulated MC	↓CTGF, S1PR2, and S1PR3	Zhong et al. (2015)
Qilong-Lishui granule	Tonifying Qi, removing stasis, removing dampness, and promoting diuresis	Bupleuri Radix, Astragali Radix, Angelicae Sinensis Radix, Dioscoreae Nipponicae Rhizoma, Polyporus, and Pyrrosiae Folium	PAN-NS rats	↓BMPRII and Smad1	Li et al. (2007)
HuangQi decoction	Nourishing Qi and Yin and tonifying the spleen and kidneys	Astragali Radix, Poria, Rehmanniae Radix, Trichosanthis Radix, Ophiopogonis Radix, Schisandrae Chinensis Fructus, and Glycyrrhizae Radix et Rhizoma Praeparatacum Melle	UUO mice and TGF-β1-stimulated HK2 cells	↓TGF-β1, TβRI, TβRII, Smad4, Smad2/3, Wnt3/4, Frizzled4, and LRP5/6; ↑GSK-3β, Axin, APC, CK1, and E-cadherin	Jiang et al. (2015); Zhao et al. (2016)
Danggui Shaoyao San	Invigorating blood circulation and promoting diuresis	Angelicae Sinensis Radix, Paeoniae Radix Alba Rhizoma, Chuanxiong Rhizoma, Atractylodis Macrocephalae Rhizoma, Alismatis Rhizoma, and Poria	ADR-NS rats and Ang II-stimulated MPC-5 cells	↑Nephrin; ↓AT1R, TRPC6, and caspase-3	Man-Man Li et al. (2021)
Chailing decoction	Smoothing liver, strengthening the spleen, promoting diuresis, and removing turbidity	Bupleuri Radix, Scutellariae Radix, Pinelliae Rhizoma, Ginseng Radix et Rhizoma, Glycyrrhizae Radix et Rhizoma, Zingiberis Rhizoma Recens, Jujubae Fructus, Polyporus, Cinnamomi Ramulus, Atractylodis Macrocephalae Rhizoma, Poria, and Alismatis Rhizoma	CsA-NS rats	↓α-SMA, TGF-β1, and Col III	Wang et al. (2011)
Jianpi qinghua recipe	Invigorating the spleen, improving Qi, clearing heat, and removing dampness	Codonopsis Radix, Astragali Radix, Tsaoko Fructus Semen, Atractylodis Rhizoma, Coptidis Rhizoma, and Prepared Rhei Radix et Rhizoma	ADR-NS rats	↓α-SMA, Col III, FN, Col IV, IL6, and CD4^+^/CD8^+^	Ma and He, (2014a); Ma and He, (2014b)
Shenhua tablet	Tonifying Qi, nourishing Yin, activating Blood, and removing stasis	Astragali Radix, Ligustri Lucidi Fructus, Atractylodis Macrocephalae Rhizoma, Paeoniae Radix Alba Rhizoma, Sparganii Rhizoma, Curcumae Rhizoma, and Lonicerae Japonicae Flos	Anti-Thy-1 nephritis rats, IRI rats, and 5/6 Nx rats	↓p-Erk1/2, cyclin D1, TLR2, TLR4, MyD88, TNF-α, and IL-6; ↑p21	Geng et al. (2016); Qing-Ping Li et al. (2019)
Jian-Pi-Yi-Shen formula	Fortifying the spleen, tonifying the kidneys, activating Blood, and removing stasis	Astragali Radix, Atractylodis Macrocephalae Rhizoma, Dioscoreae Rhizoma, Cistanches Herba, Amomi Fructus Rotundus, Salviae Miltiorrhizae Radix et Rhizoma, Rhei Radix et Rhizoma, and Glycyrrhizae Radix et Rhizoma Praeparatacum Melle	5/6 Nx rats	↑Bcl-2, HO-1, and Nrf2; ↓TGF-β, Col I, Col II, Col III, Bax, caspase 3, caspase 9, TNF-α, IL-1β, IκBα, NF-κB p65, MCP-1, CXCL1, COX-2, iNOS, and Keap1	Zhou et al. (2021)
Kangxianling	Strengthening Qi and removing stasis	Salvia Miltiorrhizae Radix et Rhizoma, prepared Rhei Radix et Rhizoma, Achyranthis Bidentatae Radix, Persicae Semen, and Angelicae Sinensis Radix	5/6 Nx rats and Ang II-stimulated HBZY-1 cells	↓JNK, TGF-β, Col-I, and FN	Ji et al. (2019)

#### Zhenwu Tang for Warming Kidney/Spleen Yang to Promote Diuresis

Zhenwu Tang (ZWT) is a classical CMM formula originally from an ancient medicinal book “Treatise on Febrile Diseases.” It is composed of Aconiti Lateralis Radix Praeparata (Fuzi), Poria, Atractylodis Macrocephalae Rhizoma (Baizhu), Paeoniae Radix Alba (Baishao), and Zingiberis Rhizoma Recens (Shengjiang). ZWT has been widely used as a remedy for CKD, relieving the clinical manifestations of edema, dyuria, and oliguria through warming Kidney/Spleen Yang to promote diuresis. Clinical studies have indicated that ZWT improved the clinical curative effect, alleviated the scores of symptoms such as the limb chills, decreased proteinuria, and blood lipids, improved renal function, and alleviated serum levels of IL-8, IL-13, CTGF, and hepatocyte growth factor (Tian et al., 2017).

The warming Yang effect of ZWT is related to antiinflammation and antifibrosis. In improving the inflammatory response, ZWT significantly reduced the serum levels of AGEs and decreased the release of inflammatory mediators (TNF-α, IL-1β, and IL-6) (Wu J. et al., 2016). The mechanism is closely related to upregulating the expression of peroxisome proliferator-activated receptor-gamma (PPAR-γ) protein and IκB mRNA, downregulating the expressions of RAGE1, p-p65, and p-IκBα, and inhibiting the activation of the NF-κB/NLRP3 pathway (Wu J. et al., 2016; Liu et al., 2018; Liu et al., 2019; Li H. et al., 2020). Interestingly, ZWT blocked the activation of NLRP3 inflammasome and inhibited IL-1β and caspase-1, which is closely related to dramatically promoting the secretion of exosomes in renal tissues (Li H. et al., 2020). In ameliorating renal fibrosis, ZWT significantly inhibited renal mRNA and protein expression of Wnt4 and its downstream genes β-catenin and Axin (La et al., 2018). In addition, ZWT could ameliorate streptozotocin-induced proteinuria and podocyte injury by suppressing the hyperactivity of the renal renin–angiotensin system and modulating the slit diaphragm (Cai et al., 2010).

The diuretic effect of ZWT was tested in NS rats and kidney-Yang-deficient rats. It turns out that ZWT could attenuate ADR-induced renal edema, which was related to the downregulation of miR-92b expression and upregulation of AQP2 expression (Liang et al., 2019). The rat model of kidney-Yang deficiency was made by injecting hydrocortisone acetate. The results showed that ZWT could adjust the osmotic pressure set point, increase the secretion of plasma aldosterone, and reduce the secretion of antidiuretic hormone, thereby promoting the excretion of Na^+^ and K^+^ and keeping the balance of water and the electrolyte content (Liang et al., 1999).

#### YiQi QingRe Gao for Benefiting Qi, Consolidating the Superficial Resistance, Heat Clearing, and Detoxifying

According to the characteristics of chronic nephritis patients, such as kidney deficiency, spontaneous sweating, and susceptibility to exogenous evil, Zhan and Dai put forward the treatment of invigorating Qi and consolidating the superficial resistance (supportive treatment); on the other hand, patients with chronic nephritis are often combined with the exogenous wind-heat syndromes, damp-heat syndromes, and blood stasis syndromes, which should be treated by clearing heat and removing dampness, as well as promoting blood circulation and removing blood stasis (Zhan et al., 2003). Therefore, YiQi QingRe Gao (YQQRG), a CMM formula, was developed and has been applied in Guang’anmen Hospital for more than two decades.

As an empirical formula, YQQRG is composed of 12 CMMs. The prescription takes AR as the monarch medicine, combined with Atractylodis Macrocephalae Rhizoma and Saposhnikoviae Radix (Fangfeng), which is the composition of Yupingfeng San, to invigorate Qi, consolidate the superficial resistance, and eliminate evil or toxin in the outside; Lonicerae Japonicae Flos (Jinyinhua), Forsythiae Fructus (Lianqiao), Duchesneae Indicae (Shemei), Hedyotis Diffusa (Baihuasheshecao), and Imperatae Rhizoma (Baimaogen) can clear heat and remove dampness in the inside; Poria, Alismatis Rhizoma (Zexie), Leonuri Herba (Yimucao), and Dioscoreae Nipponica Rhizoma (Chuanshanlong) can promote blood circulation and diuresis and guide the evil of dampness, heat, and turbid to go out. In a previous clinical study, it was found that YQQRG could significantly reduce TCM symptom scores of patients with chronic glomerulonephritis, effectively decrease 24 h urine protein, and improve plasma albumin and immunoglobulin (Ig) G, IgA levels, and the effective rate of treatment was 90% (Zhan and Dai, 2003).

Animal experiments have shown that the formula inhibits the expression levels of inflammatory factors such as TNF-α, TNFR1, IL-1β, and MCP-1 in renal tubulointerstitium, downregulates the expression level of caspase-3 in the renal tissues, and upregulates the expression level of Bcl-2 to exert an antiapoptotic effect (Wen et al., 2015). In maintaining the glomerular filtration barrier, YQQRG could upregulate the expressions of podocyte-related proteins such as nephrin, podocin, and CD2AP in kidney tissues of rats with PAN nephropathy and inhibit their mRNA level in feedback (Zhan et al., 2014). Moreover, the main molecular chaperones of endoplasmic reticulum stress, such as glucose-regulated protein 78 (GRP78) and GRP94, and cytoskeletal regulatory proteins, such as α-actin-4, synaptopodin, desmin, and uPAR, were all inhibited by YQQRG (Yang et al., 2016a). An *in vitro* experiment indicated that treatment with serum-containing YQQRG inhibited LPS-induced rat mesangial cell proliferation, downregulated mRNA and protein levels of Wnt4 and TGF-β1, and reduced the aggregation of the mesangial matrix (Yang et al., 2016b). Dioscin might be the key component in YQQRG to exert the above effects.

#### Qufeng Tongluo Recipe for Expelling Wind, Dredging Collaterals, Invigorating Qi, and Tonifying the Kidneys

Sun et al. consider that the pathogenesis of CKD is the deficiency of kidney collaterals caused by wind pathogen, mixed with blood stasis and dampness heat (Sun et al., 2008). Therefore, Qufeng Tongluo Recipe (QTR) was developed as an empirical formula. This prescription selects Zaocys (Wushaoshe) as the monarch medicine and Sinomenii Caulis (Qingfengteng) and Piperis Kadsurae Caulis (Haifengteng) as the minister medicines, and it takes the properties of insects’ moving and dredging collaterals (dredging the channel) and vines’ winding and spreading to achieve the effects of dispelling wind, eliminating dampness, and dredging collaterals. This is assisted by AR to invigorate Qi, dredge collaterals, promote diuresis, and reduce swelling and Taxilli Herba (Sangjisheng) to nourish the liver and kidneys and dispel wind and dampness. Rehmanniae Radix (Dihuang) is used as the courier to nourish Yin and generate fluid, which not only prevents the dryness of the herbs but also leads the herbs straight into the kidney meridian. All the CMMs are combined to achieve the effects of expelling wind, dredging collaterals, invigorating Qi, and tonifying the kidneys. In the clinical treatment of DN-complicated CRF, QTR showed the effect of reducing blood lipid, improving renal function, and reducing serum TGF-β1 level (Sun et al., 2004).

Animal experiments showed that QTR could improve kidney injury by regulating the expressions of cytoskeletal proteins (synaptopodin, α-actinin-4, and desmin) and slit diaphragm proteins (CD2AP) (Wang Z. et al., 2014a; Wang Z. et al., 2014b), increasing the anion site and HSPG expression in the GBM to protect the basement membrane damage (Sun et al., 2010; Ma et al., 2011). In addition to improving the glomerular filtration function, QTR could also inhibit the accumulation of ECM by reducing the expressions of Col IV, fibronectin, and laminin in kidney tissues and downregulate the expression of renal fibrosis-related proteins α-SMA and vimentin, thus playing an antifibrosis role (Wu et al., 2008; Ma et al., 2019). The results *in vitro* indicated that the inhibition of QTR on the proliferation of mesangial cells was related to the regulation of the cell cycle process and the inhibition of TGF expression (Sun et al., 2008; Wu et al., 2013). The regulation of cell cycle progression is reflected in the reduction of the protein and mRNA expression levels of cylinD1, cyclin-dependent kinase 2 (CDK2), and p21 and the increase of p27.

#### Uremic Clearance Granule for Invigorating the Spleen, Dispelling Dampness, Removing Turbidity by Catharsis, Promoting Blood Circulation, and Removing Stasis

Uremic clearance granule (UCG) is the first CMM preparation in the field of treating early and mid-stage CRF. It contains 16 kinds of CMMs and exerts the effects of removing turbidity by catharsis, invigorating the spleen, dispelling dampness, promoting blood circulation, and removing blood stasis in TCM. In some small-scale clinical trials, UCG is effective in reducing serum creatinine levels, improving renal blood flow, and improving the state of microinflammation (Wu et al., 2004). In a multicenter clinical trial involving 300 patients with stage 3b-4 CKD, UCG significantly reduced serum creatinine, delayed the reduction of estimated GFR, and delayed the progression of CKD at 24 and 48 weeks after administration (Zheng et al., 2017; Zheng et al., 2019). Subsequently, it was recommended in the clinical application guide of Chinese patent medicine in the treatment of CKD stage 3–5 (nondialysis), version 2020 (The Standardization Project Team of The Clinical Application Guide of Chinese Patent Medicine in The Treatment of Dominant Diseases, 2021).

In the clinical application of UCG in the recent 30 years, its mechanism of action has been comprehensively studied. In terms of inhibiting the tubular epithelial to mesenchymal transition, UCG could significantly increase E-cadherin expression and suppress vimentin and α-SMA expression both *in vivo* and *in vitro* (Lu et al., 2013). On the effect of oxidative stress, UCG showed the effect of restoring mitochondrial regeneration (shown as restoring the expression of mitochondrial transcription factor A and peroxisome proliferator-activated receptor γ co-activator-1α), improving mitochondrial structure and function, and further inhibiting the apoptosis of tubular epithelial cells, which is related to the inhibition of the TGF-β1-ROS-MAPK pathway (Xu et al., 2015). Focusing on the role of TGF-β1 expression in renal fibrosis, it was proved that UCG downregulated the protein expressions of TGF-β1, TGF-β receptor I, receptor II and Smad2/3, and upregulated the protein expressions of SnoN and Smad7 (Huang et al., 2014; Wu W. et al., 2016). Further experiments revealed that regulation of TGF-β1 transcription and translation by UCG was due to induction of remethylation of the TGF-β1 promoter in CRF rats (Miao et al., 2010). In addition, the effect of UCG in correcting the imbalance of MMP-2/TIMP-1 in model rats has also been elucidated (Huang et al., 2014).

With the continuous research on UCG, the original prescription was simplified and recombined, and finally the second-generation product Huang Gan formula (HGF) was formed. HGF is composed of RRR, Zingiberis Rhizoma, Bupleuri Radix, Glycyrrhizae Radix et Rhizoma, and Aconiti Lateralis Radix Preparata. It can significantly improve the renal function of rats in CRF models, reduce oxidative stress injury, and delay the development of renal fibrosis (Xiao et al., 2014). At present, the mechanism of HGF is relatively limited. Mo et al. showed that HGF plays an antiinterstitial fibrosis role by inhibiting the Wnt/β-catenin pathway through depressing protein and mRNA expressions of Wnt1, β-catenin, transcription factor 4, and FN (Mo et al., 2015). Similar to UCG, HGF also shows the effect of antioxidative stress. The research indicated that the effect of HGF in advanced oxidation protein products-induced human mesangial cells was related to the inhibition of the JAK2/STAT3 pathway and the regulation of the balance of the advanced glycation end products receptor (Deng et al., 2017).

**FIGURE 2:**
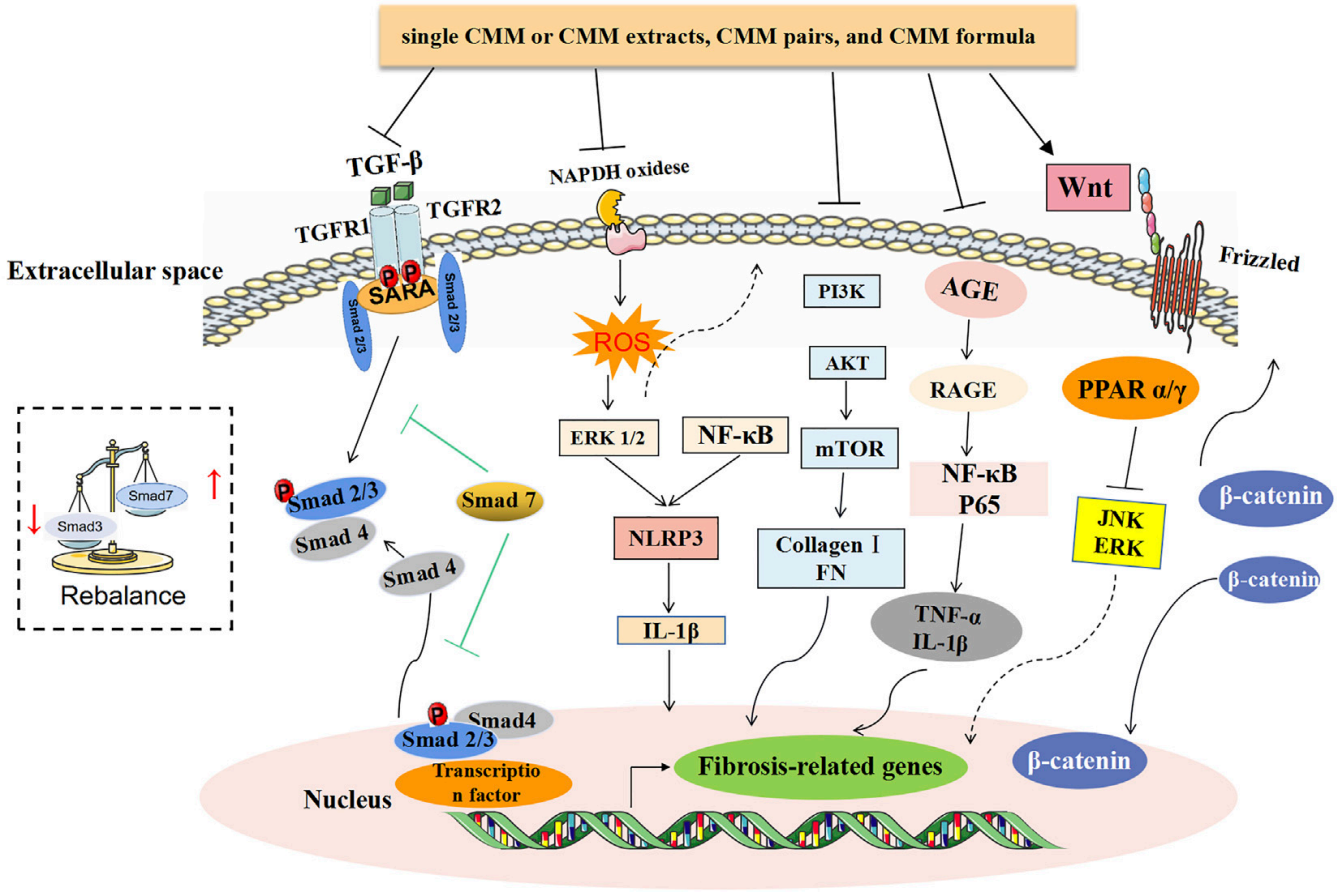
Summary of the related mechanism of treating CKD with CMM. TGF-β/Smad and Wnt/β-catenin signaling pathways are two antifibrotic mechanisms that have been confirmed by a variety of TCM studies. Among them, Smad3 is pathogenic in renal fibrosis but Smad7 plays a protective role by negatively regulating the phosphorylation of Smad2/3 and NF-κB-driven inflammatory response. For the dual functions of Smad4, the therapeutic effect of CMM is to inhibit Smad3-dependent renal fibrosis. Besides, the sustained excessive reaction of abnormal activation of the PI3K/Akt/mTOR signaling pathway can lead to an increase in Col I and FN and ultimately lead to renal fibrosis. In the occurrence of glomerular hyperperfusion, hyperfiltration, and hyperglycemia, it causes oxidative stress and AGE activation, followed by a series of inflammatory reactions. In addition to inhibiting the activation of AGE, the antiinflammatory effect of CMM is also manifested in the inhibition of ROS-ERK1/2 and NF-κB-mediated NLRP3 inflammasomes.

The above systematically summarizes the research strategies and mechanisms of TCM in treating CKD (the relevant mechanisms are summarized in Figure 2). At present, many small clinical studies have been published in Chinese journals to support the kidney protective effect of TCM on CKD, and there are many systematic reviews and meta-analyses. With the gradual deepening of research, more and more large-scale well-designed randomized controlled trials have been carried out. Table 2 summarizes the large-sample clinical trials on the prevention and treatment of various CKD with TCM in recent years, which further supports the effectiveness of TCM.

**TABLE 2:** Recent trials studying the use of TCM in CKD.

TCM intervention	Disease	*N*	Trial period (mo)	Primary outcome	Outcome	Ref.
Shenqi particle vs. prednisone/cyclophosphamide	Idiopathic membranous nephropathy	190	12	CR + PR	46/63 (73.0%) vs. 54/69 (78.3%); *p* = 0.5	Chen et al. (2013)
Bupi Yishen formula vs. losartan	Nondiabetic CKD stage 4	567	12	eGFR slope	−2.3 vs. −4.5 ml/min/1.73 m^2^/year (*p* < 0.05)	Mao et al. (2021)
Tangshen formula vs. placebo	Type 2 diabetic kidney disease	180	6	UAER + 24 h UP	UAER: −19.5 vs. −7.0 μg/min (*p* = 0.7); 24 h UP: −0.2 vs. 0.4 g (*p* < 0.05)	Li et al. (2015)
TCMsa vs. benazepril vs. TCMs/benazepril combination	CKD stage 3	578	6	Mean eGFR	48.5 ± 15.9 vs. 43.0 ± 12.4 vs. 48.3 ± 17.5 ml/min (*p* < 0.05)	Yong-jun Wang et al. (2012)
Niaoduqing particles vs. placebo	CKD stage 3b-4	300	6	Changes in Scr and eGFR between pre treatment and posttreatment	Scr: 1.1 vs. 11.7 μmol/L (*p* < 0.01); eGFR: −0.2 vs. −2.2 ml/min/1.73 m^2^ (*p* < 0.05)	Zheng et al. (2017)
Zicuiyin decoction vs. Huangkui capsule	Diabetic kidney disease	88	2	Changes in eGFR between pre treatment and posttreatment	2.6 ± 18.5 vs. −7.1 ± 24.7 ml/min/1.73 m^2^ (*p* < 0.05)	Liu et al. (2022)
Huangkui capsule vs. losartan	IgA nephropathy	1470	12	Changes in 24 h UP between pre treatment and posttreatment	−230 vs. −253 mg (*p* = 0.6)	Li P. et al. (2020)
Chinese herbal formula granules vs. placebo	CKD stage 3	343	6	24 h UP + Scr + eGFR	24 h UP: 1.0 ± 1.1 vs. 1.0 ± 1.3 g/d (*p* > 0.05); Scr: 130.8 ± 32.6 vs. 149.1 ± 41.3 μmol/L (*p* < 0.05); eGFR: 55.7 ± 50.8 vs. 44.5 ± 12.6 ml/min/1.73 m^2^ (*p* < 0.05)	Zhao et al. (2020)
TCMsa vs. losartan	CKD stage 1–2	396	6	24 h UP	801.6 ± 911.7 vs. 1080.6 ± 925.8 mg/L (*p* < 0.05)	Wu et al. (2015)
Huangkui capsule vs. losartan vs. Huangkui capsule/losartan combination	Primary glomerular disease	417	6	Changes in 24 h UP between pre treatment and posttreatment	−508 vs. −376 mg/d (*p* = 0.003) vs. −545 mg/d (*p* < 0.001)	Zhang et al. (2014)
TCMsa vs. losartan	CKD stage 1–2	81	6	24 h UP	0.4 ± 0.6 vs. 0.2 ± 0.9 g (*p* < 0.05)	Wang et al. (2013)
Qidan Dihuang grain/angiotensin receptor blocker combination vs. angiotensin receptor blocker	Diabetic kidney disease	102	3	24 h albuminuria	41.4 vs. 47.7 mg (*p* < 0.05)	Xiang et al. (2016)
Shenhua tablet vs. fosinopril	IgA nephropathy	131	3	24 h UP + TCM syndrome score	24 h UP: 1.05 ± 1.07 vs. 1.13 ± 1.18 g (*p* > 0.05); TCM syndrome score: 2.86 ± 2.06 vs. 3.53 ± 1.91 (*p* < 0.05)	Chen et al. (2007)

## Discussion and Perspectives

CKD is a common and progressive disease that has become a major public health problem on a global scale. However, the single target characteristic of modern medicine cannot meet the clinical requirements. According to the syndrome differentiation of diseases, CMM with multilink and multipath effects is selected, which is indeed effective for complex diseases. Treatment based on syndrome differentiation is a very important feature of TCM. The syndrome is a summary of the body’s pathological response at a certain stage in the development of the disease, including the location, cause, nature of the lesion, and relationship between healthy Qi and pathogenic factor, reflecting the essence of pathological changes at this stage (Chen and Lu, 2006). Only when doctors accurately identify the TCM symptoms of the disease can they issue an accurate prescription. Therefore, treatment based on syndrome differentiation comprehensively considers the various factors of the disease and provides a multilink and multipath control method for the prevention and treatment of CKD. In addition, under the guidance of syndrome differentiation and treatment, the prescriptions used by TCM physicians also conformed to the compatibility theory of “Jun-Chen-Zuo-Shi,” which clearly pointed out the roles played by each ingredient in the prescription (Luan et al., 2020). Under the guidance of these rules, TCM emphasizes the treatment of the variable related to systemic disease and different targets and allows some ingredients in prescriptions to be changed when symptoms and signs change, so TCM presents an ideal choice for the intervention of chronic diseases.

Modern medicine considers that in the course of the disease, renal fibrosis is a general characteristic of CKD. Although there is no “renal fibrosis” in the TCM theory, the progressive development of various kidney diseases can be manifested as a deficiency in the root and excess in the branch, and the change of syndromes can occur, such as “edema,” “retention of urine,” and “kidney fatigue” in TCM. This is consistent with the understanding of the disease process in modern medicine. The ancients believed that “most of the kidney diseases are of deficiency syndrome.” Modern physicians also agree that kidney deficiency should be considered as the pathological basis of kidney disease and even believe that there is no kidney disease without kidney deficiency (Liu et al., 2011a). Together with the views that kidney-Qi deficiency is a leading cause of renal fibrosis (Cao et al., 2015; Zhao, 2019), it is considered that the pathogenesis of root deficiency may be reflected in renal fibrosis. As summarized in this study, many single CMM and CMM formulas aiming at the pathogenesis of kidney deficiency of CKD have different compositions but all exhibit antifibrosis effects. In addition, these CMMs can improve renal fibrosis in different disease models (such as NS, UUO, 5/6Nx, and CRF), which embodies the theory of “same treatment for different diseases” in TCM. For example, ZWT can be used to treat DN, NS, CRF, and other diseases by improving renal fibrosis as described above. Since CMMs have antiinflammatory, antioxidation, antiapoptosis, and antifibrosis properties and play a role in the regulation of immunity, they can inhibit the TGF-β1/Smad, NF-κB, PI3K/Akt/mTOR, Wnt/β-catenin, ERK-1/2, p38 MAPK, JNK, and other pathways and activate the HGF/c-met pathway to exert an antifibrosis role.

In the process of the progression of CKD, in addition to the common pathology of renal fibrosis, it is also related to the infiltration of inflammatory factors, renal ischemia injury, hyperlipidemia, and the change of blood volume (Peng et al., 2005; Dou et al., 2009; Liu, 2011). Here, due to the reduced renal filtration function in CKD, a large number of potentially nephrotoxic components (such as complement factors, growth factors, and others) are abnormally filtered into the urine, causing these macromolecules to damage the tubulointerstitium, and these substances induce renal interstitial damage far more serious than that of urine albumin (Cravedi and Remuzzi, 2013). Therefore, causing proteinuria has a certain toxic effect. As mentioned above, the pathogenic excess in the pathogenesis of CKD mainly includes blood stasis, dampness heat, turbid toxin, and water stagnation. Therefore, both modern medicine and TCM can recognize the different characteristics in the pathogenesis of CKD, and their understanding of the characteristics of the disease can be explained by each other. Briefly, the microscopic manifestations of blood stasis can be described as the disorder of local blood microcirculation in the kidneys, resulting in ischemia and hypoxia of kidney tissues, necrosis, and apoptosis of endothelial cells as well as ischemic collapse and sclerosis of glomerular (Guo et al., 2019). The microscopic manifestations of dampness heat are the lesion of the capillary loop, the infiltration of interstitial inflammatory cells, the proliferation of glomerular cells, and segmental sclerosis or adhesion of glomerulus (Yu et al., 1992; Wang et al., 2008; Wang X. et al., 2015). Turbid toxin manifests as the abnormal increase of solutes with different molecular weights, such as inflammatory factors, urine protein, LDL, and oxidized LDL (Vanholder et al., 2003; Palmer, 2007). Water stagnation can be manifested as an abnormal increase in blood volume, leading to edema (Rodríguez-Iturbe et al., 2002; Ellison, 2017). Therefore, when blood stasis is present, SM or Shenkangling, which promotes blood circulation and removes blood stasis, is used to improve the expressions of nNOS, HIF-1α, and VEGF. When NS is accompanied by dampness heat syndrome, AC or Wulingsan, which has the effect of clearing dampness heat, can be selected to reduce the infiltration of inflammatory cells and the proliferation of glomerular cells. Likewise, ZWT, which regulates the distribution and expression of AQP2, can be used to eliminate edema and UCG, which inhibits the apoptosis of renal tubular epithelial cells and reduces the toxicity of proteinuria, can be used to reduce the turbidity. To sum up the above points, the use of different treatments at different stages of disease development is the idea of “different treatments for the same disease” in TCM.

It should be pointed out that in the course of disease development, different pathogenic factors can influence each other. Just as the infiltration of inflammatory cells is the microscopic manifestation of dampness heat, it is also the initiating factor of fibrosis. Besides, if the dampness heat is not removed, it will lead to the accumulation of solutes with different molecular weights and the formation of turbid toxin. Therefore, TCM can not only regulate the root of the disease (renal fibrosis), but also regulate other factors toward normal from different links and the balance of Yin and Yang.

However, there are still some issues that need to be resolved. *1*) The current clinical evidence, even from the meta-analysis, is not enough to support the clinical application of CMMs in kidney disease. More large-scale clinical trials are needed to assess the efficacy, effectiveness, and toxicity of CMMs to optimize their therapeutic and preventive potential. *2*) The therapeutic effects of CMMs are attributed to their unique multitarget and multipath effects that are difficult to separate from the different stages of CKD. Therefore, when to use CMM and what kind of efficacy of CMM should be selected mainly depend on clinicians’ self-experience. It is urgent to construct modern pharmacological indicators to characterize the conditions for the application of CMMs, to facilitate the globalization of the application of CMMs. *3*) Different prescriptions of TCM have different therapeutic characteristics, but there are few specific indicators that can reflect the action characteristics of TCM. It is necessary to clarify the scientific connotation of TCM with clear pharmacological indexes. *4*) The multitarget mechanism of the TCM therapy has not been fully elucidated, and most of them are based on a single pathway. However, the interaction relationship between different signaling pathways is not clear yet, and the cross-targets among the pathways are rarely studied. It is necessary to deeply explore the mechanism and the interaction relationship of TCM in a multidisciplinary way and visualize the multitargets and multilink network of CMMs. *5*) Currently, some ingredients derived from CMMs also show good efficacy, but it is unclear which ingredient has the best efficacy or which one is the most effective among CMMs and their ingredients. A well-designed randomized controlled trial is needed to confirm the efficacy and safety of potential drugs.

## Conclusion

In summary, TCM has made major progress in the treatment of CKD and has shown a beneficial role in delaying CKD in clinical, animal, and *in vitro* studies. The effectiveness of CMM is the result of long-term clinical practice under the guidance of the TCM theory, which seeks to “reinforce deficiency and purge excess” through the synergistic effect of multitargets and multipaths. CMMs can not only inhibit the activation of myofibroblasts, the infiltration of inflammatory cells, the transdifferentiation of epithelial cells to mesenchymal cells, and the excessive deposition of ECM to improve renal fibrosis through various mechanisms, but also improve blood flow, inhibit the proliferation of glomerular cells and the accumulation of solutes, and relieve water retention to achieve a relative balance between Yin and Yang, which highlights the advantages of holistic control of TCM and provides a good therapeutic strategy for clinical treatment of CKD. Further research should focus more attention to large-scale clinical trials and combine the TCM theory with the latest medical research to deeply explore its mechanism, which is of great significance for TCM treatment of CKD.

## Data Availability

The raw data supporting the conclusions of this article will be made available by the authors, without undue reservation.
